# Continuation of Pregnancy After Donor Twin Demise Following Fetoscopic Laser Photocoagulation to Treat Twin-Twin Transfusion Syndrome

**DOI:** 10.7759/cureus.84544

**Published:** 2025-05-21

**Authors:** Shravani Rama, Sanjana D Nalla, Ashwin R Jadhav, Constantino G Lambroussis

**Affiliations:** 1 Obstetrics and Gynecology, Gandhi Hospital, Hyderabad, IND; 2 Obstetrics and Gynecology, Lifeline Medical Associates, Edison, USA; 3 Medicine, Lake Erie College of Osteopathic Medicine, Erie, USA; 4 Obstetrics and Gynecology, Saint Peter’s University Hospital, New Brunswick, USA; 5 Osteopathic Medicine/Family Medicine, Lake Erie College of Osteopathic Medicine, Erie, USA

**Keywords:** congenital pulmonary airway malformation, donor twin demise, fetoscopic laser photocoagulation, monochorionic diamniotic pregnancy, twin-twin transfusion syndrome

## Abstract

Monochorionic diamniotic (MCDA) pregnancy with eccentric cord insertion may lead to twin-twin transfusion syndrome (TTTS), which can be treated by fetoscopic laser photocoagulation (FLP). The procedure has its risks, including the demise of one or both twins. A 33-year-old primigravida woman with MCDA twins was diagnosed with TTTS and eccentric cord insertion at 18 weeks of gestation. FLP was performed, resulting in the demise of the donor twin. The surviving twin was later diagnosed with congenital pulmonary airway malformation and was delivered at 38 weeks with stable outcomes, while the papyraceous twin was delivered by manual extraction. This case discusses eccentric cord insertion as a possible cause of TTTS managed with FLP and its associated risks. It emphasizes the necessity for rigorous surveillance and timely interventions in high-risk pregnancies, showcasing the importance of a comprehensive care approach.

## Introduction

Multiple pregnancies are a prevalent complication of assisted reproductive techniques. To mitigate this, elective single embryo transfer (eSET) is used along with preimplantation genetic testing (PGT), where genetically normal embryos are implanted, enhancing the likelihood of successful conception [1]. About 1.36% of single-embryo transfers may result in twinning; 0.9-1.3% are monozygotic twins, and 0.048% are triplets [2]. Monochorionic diamniotic (MCDA) twin pregnancies pose risks, notably twin-twin transfusion syndrome (TTTS), which results from imbalanced blood flow between donor and recipient twins, potentially causing severe outcomes if untreated. In MCDA twin pregnancies, discordant CRL greater than 10.0% was related to fetal growth restriction. Meanwhile, an intertwin discordance of nuchal translucency thickness greater than 20.0% was unrelated to TTTS, fetal growth restriction, and intrauterine fetal demise. However, adequate surveillance is still required [3]. Fetoscopic laser photocoagulation (FLP) therapy is the standard for stage 2 TTTS, addressing abnormal vascular connections and improving outcomes. However, complications such as the potential death of either twin may still occur post-therapy [4]. This case report discusses the management of a MCDA twin pregnancy after FLP therapy for TTTS.

## Case presentation

A 33-year-old primigravida woman conceived via in vitro fertilization after eSET and PGT for aneuploidies. At seven weeks and two days, it was confirmed to be a MCDA twin pregnancy. Prenatal labs, such as complete blood count, urinalysis, and infection screening, were unremarkable except for a negative Rhesus factor status. The medical history was significant for hypothyroidism. Surgical history was remarkable for knee surgery, hysteroscopy, and oocyte retrieval. The patient was on prenatal vitamins and 25 mcg of levothyroxine throughout her pregnancy.

**Figure 1:**
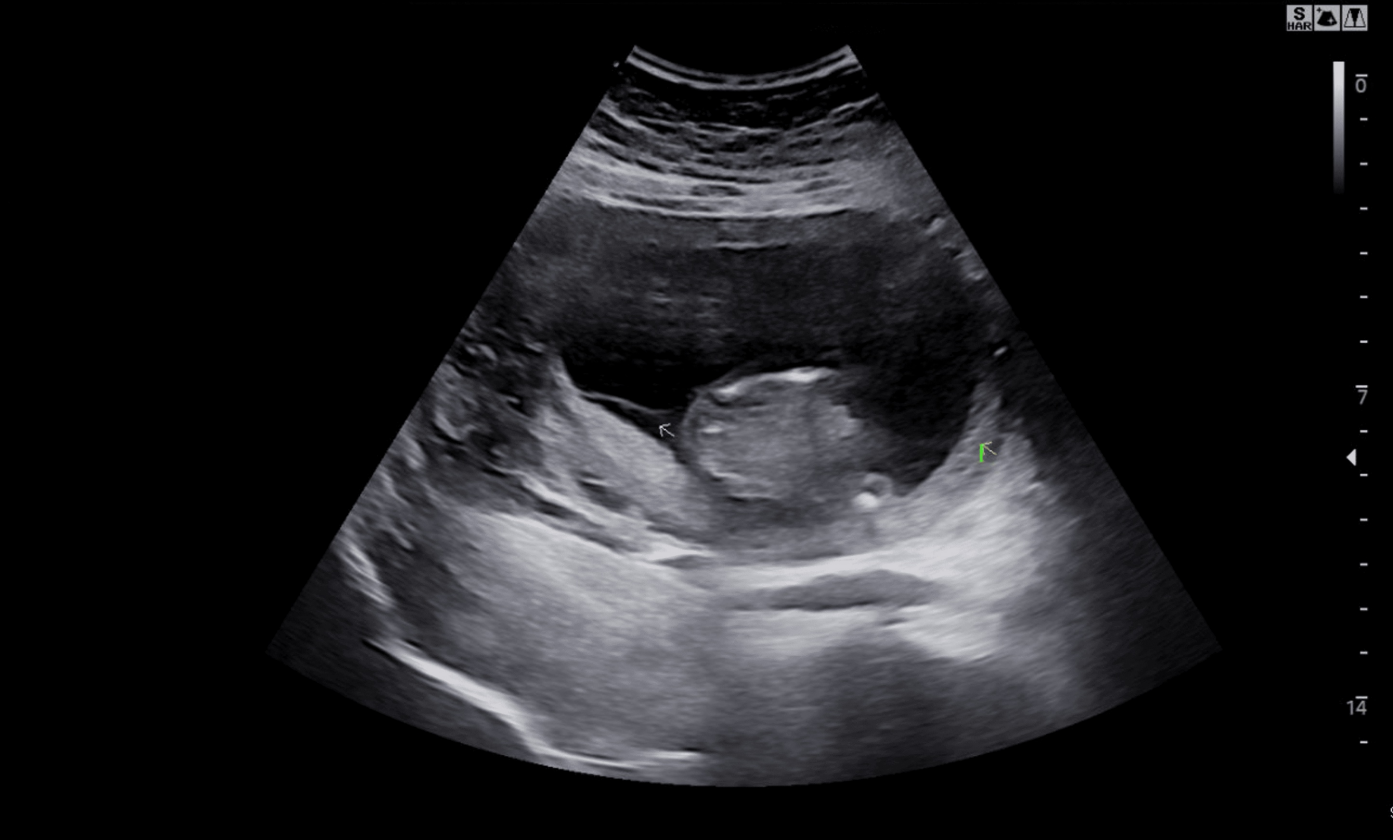
Membrane separating the amniotic sacs in our 33-year-old primigravida woman (arrow)

**Figure 2:**
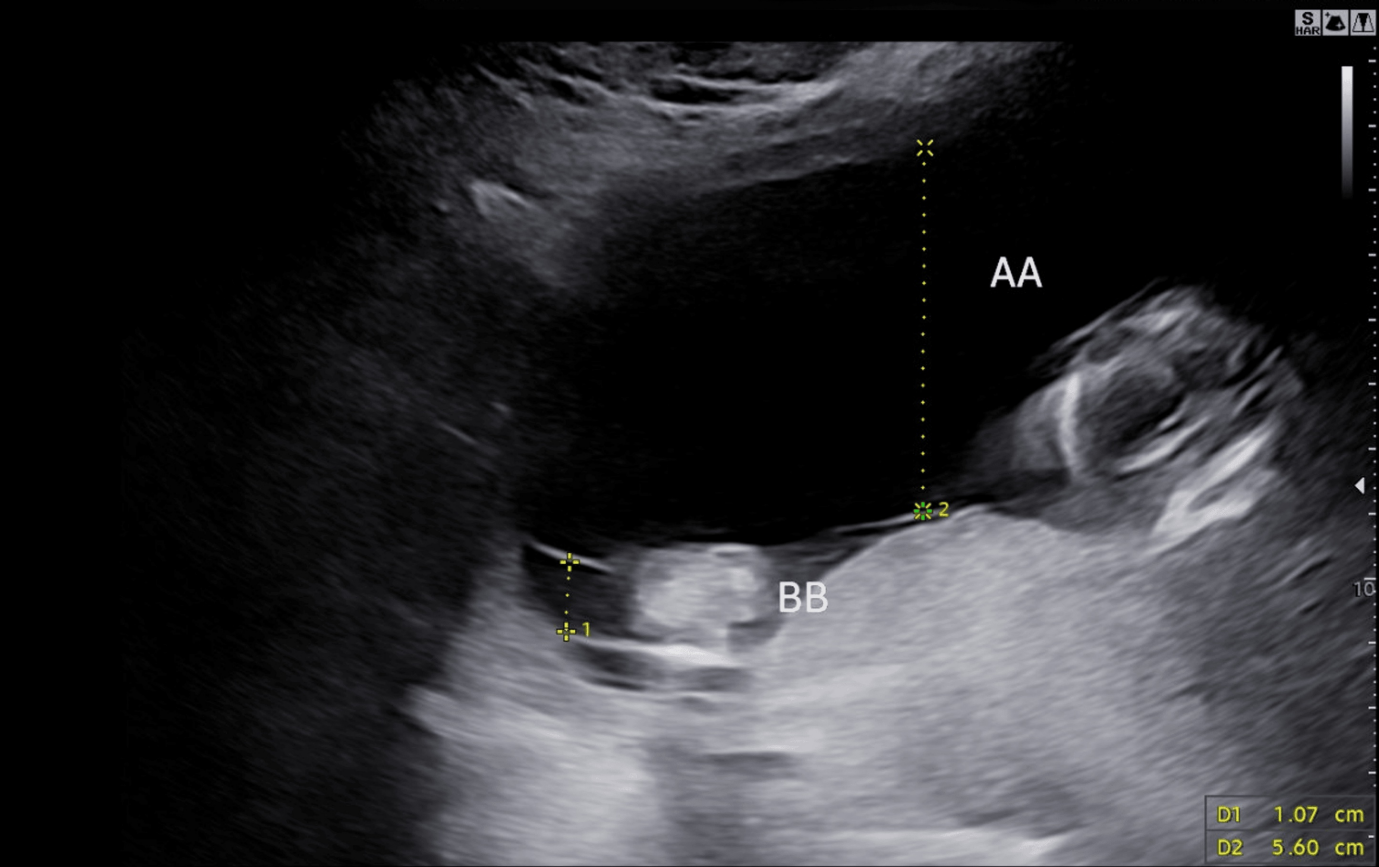
Scan showing the difference between amniotic fluid levels at 16 weeks and one day in our 33-year-old primigravida woman

At 16 weeks and one day, ultrasound showed twin A (recipient twin) with normal amniotic fluid and an estimated fetal weight of 172 g, appropriate for gestational age. Twin B (donor twin) lagged by eight days and had oligohydramnios with a 27% growth discordance. Figure 1 shows the membrane separating the amniotic sacs in the patient, and Figure 2 shows the differences in amniotic fluid levels at 16 weeks and one day. The patient was counseled on the potential development of TTTS, which may result in growth restriction, oligohydramnios, or demise of the donor twin (twin B) and carries risks of heart failure, hydrops, or demise of the recipient twin (twin A). After thorough counseling, the patient was referred to the Regional Center for Fetoscopic Surgery for further evaluation and management of TTTS.

The evaluation included fetal echocardiography and high-resolution ultrasound. Echocardiography showed normal cardiac structure and function in both fetuses and a cardiac score of 5/20. High-resolution ultrasound suggested eccentric placental cord insertions with an inter-cord distance of 8.9 cm. Twin A had the deepest vertical pocket of 6.4 cm and an estimated fetal weight of 186 g (90th percentile). In contrast, twin B had the deepest vertical pocket of 1.9 cm and an estimated fetal weight of 154 g (37th percentile), representing 17% intertwin-size discordance. Middle cerebral artery Doppler studies demonstrated normal-to-elevated peak systolic velocities in both twins. The rest of the Doppler findings were appropriate for the gestational age. According to the Quintero staging system, these findings are consistent with stage 1 TTTS. Follow-up after three days showed progression of TTTS to stage 2. Management options with risks and benefits were discussed in detail.

The patient consented to undergo FLP, which was performed at 18 weeks of gestation, and RhoGam was administered post-procedure. The procedure was uneventful, except for the concern for twin B, as its placental share had minimal vasculature. Twin B’s death occurred within 24 hours after FLP. The patient and her spouse were counselled regarding the risks of demise, polyhydramnios, cardiac dysfunction, renal dysfunction, and neurological problems in the surviving twin, and she decided to continue with the pregnancy. Management continued with strict maternal-fetal monitoring, including coagulation studies of the mother and fetal surveillance. An anatomy scan at 21 weeks of gestation revealed twin A's estimated fetal weight, consistent with 22 weeks and one day of gestation: 473 g, with polyhydramnios, which was resolved in further scans.

Subsequent fetal MRI for neurological evaluation was normal but incidentally revealed a lung mass, which was confirmed during an ultrasound examination to be a 2.3 x 1.6 x 1.5 cm echogenic intrathoracic mass, including a 5 mm cyst in the inferior portion of the right lung of twin A at 27 weeks of gestation. These findings were consistent with congenital pulmonary airway malformation (CPAM) type 2, macrocystic type. Throughout the pregnancy, the mass remained stable. MRI for neurological evaluation at 32 weeks was normal.

**Figure 3:**
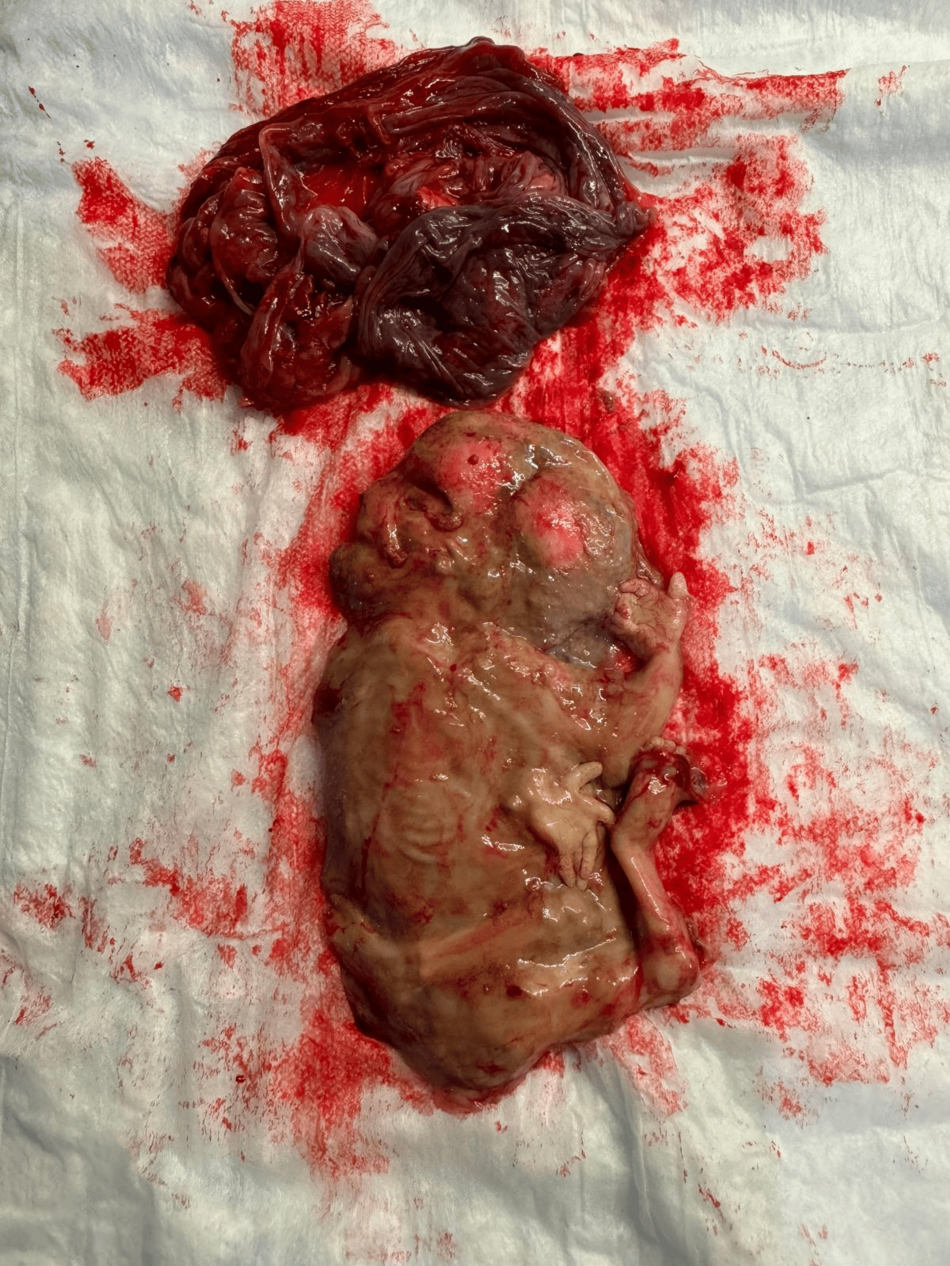
Fetus papyraceus and placenta with membranes from our 33-year-old primigravida woman

Biweekly fetal surveillance with a non-stress test, biophysical profile, and Doppler ultrasound was performed until 38 weeks of gestation. Labor was induced at 38 weeks and one day of gestation with 25 mcg of oral Cytotec. Twin A was delivered vaginally after a mediolateral episiotomy with a second-degree extension, weighing 6 lb 11 oz. Papyraceus twin B, with a sac and membranes adherent to the uterine wall, required manual extraction with an estimated blood loss of 350 mL. Perineal laceration was sutured in the usual layered form. Figure 3 shows the fetus papyraceus and placenta with membranes extracted from the patient.

Owing to the potential risk of postpartum hemorrhage, 1000 mg of misoprostol was administered rectally. The postpartum period was uneventful. The placental study revealed an eccentrically inserted cord, with the insertion located 3 cm from the nearest margin, and the membranes showed marginal insertion with mildly distended chorionic vessels with no thrombus and mild calcification. Neonatal brain MRI at six weeks postpartum was normal; however, the lung showed persistence of CPAM. Parents were counselled regarding the need for surgical management. Informed consent was obtained from the patient to mention the findings of this case report.

## Discussion

Multifetal gestations are increasingly common due to artificial reproductive techniques (ART) that transfer multiple embryos to enhance conception chances. The incidence of twin pregnancies in natural conceptions is about 1.4%, rising to 15.4% with ART [5]. eSET and PGT have reduced multiple pregnancies. However, eSET still results in twinning in 1.36% of cases, with 0.9-1.3% being MCDA twins and 0.048% being triplets [2]. MCDA twins share one placenta, requiring careful management and monitoring to ensure optimal outcomes for both fetuses. TTTS affects 10-15% of MCDA twin pregnancies and typically emerges in the second trimester [6]. The diagnosis of TTTS is debated; some studies suggest accurate diagnosis in the second trimester due to correlations between amniotic fluid levels and renal function, while others advocate for early detection based on fetal size discrepancies as early as 10 weeks of gestation. Diagnosis involves regular ultrasounds to monitor fetal growth discrepancies and amniotic fluid levels. Upon diagnosis, the severity of TTTS is assessed using the Quintero staging system. Table 1 describes the stages of the Quintero staging system [7].

**Table 1:** Quintero staging system of TTTS MVP: maximum vertical pocket, TTTS: twin-twin transfusion syndrome, A/REDV: absent or reversed end-diastolic velocity [7]

Stage	Description
Stage 1	MVP >8 cm in the recipient twin and MVP <2 cm in the donor twin
Stage 2	Visible bladder in the recipient twin and absent bladder filling in the donor twin
Stage 3	A/REDV in the donor’s umbilical artery, absent or reversed a-wave in the ductus venosus, or umbilical vein pulsations in either twin
Stage 4	Hydrops in either twin
Stage 5	Single or double fetal death

Management of stage 1 TTTS requires only close monitoring and has a 50% chance of progressing to stage 2, which is treated with FLP of shared placental vessels or amnioreduction. FLP is preferred over amnioreduction due to its lower morbidity and mortality rates [4]. FLP complications are outlined in Table 2 [8,9]. Studies indicate that 76.6% of pregnancies treated with FLP result in the survival of both twins, with donor twin demise at 9% and recipient twin demise at 7% [10]. The median interval from procedure to death is four days (1-89 days) [11]. Approximately 13% of donors die within one week post-FLP [11]. Key risk factors for donor demise include significant fetal growth discordance (>30%), reverse end-diastolic velocity in the donor’s umbilical cord, marginal or velamentous cord insertion, and a higher number of anastomoses [11]. Marginal or velamentous cord insertion in one or both twins increases the risk of adverse outcomes and TTTS [12].

**Table 2:** Complications of FLP FLP: fetoscopic laser photocoagulation, PPROM: preterm premature rupture of membranes, IVH: intraventricular hemorrhage [8,9]

Complications	%
Intraoperative bleeding at placental surface	10.5%
Preterm labor with cervical change	15.8%
Preterm uterine contractions	36.8%
Immediate PPROM	5.3%
Remote PPROM	12.3%
Iatrogenic monoamnioticity	3.5%
Neonatal morbidities	48.3%
Neurological abnormalities (IVH)	18.3%
Cardiac dysfunction	18%
Renal dysfunction	15.7%

The outcome for the surviving fetus, the dead fetus, and the mother depends on the timing of fetal death. Loss of a twin in the first trimester generally results in vanishing twin syndrome, with minimal impact on the surviving twin [13]. However, loss in the second or third trimester poses risks to both the mother and the surviving twin, including polyhydramnios and neurological complications due to hypoxic-ischemic changes. Approximately 11-20% of surviving co-twins may exhibit neurological abnormalities detectable through MRI with diffusion-weighted imaging [14], necessitating parental counseling about potential neurodevelopmental issues and antenatal and postnatal MRI evaluations. Monitoring surviving twins involves daily fetal movement counts, weekly non-stress tests, and biweekly ultrasounds with biophysical profiles and Doppler studies [13]. Although rare, maternal disseminated intravascular coagulation has been reported, warranting monitoring of maternal coagulation studies [15]. The macerated twin becomes a fetus papyraceus due to amniotic fluid and soft tissue absorption, compressed between the surviving fetus and uterine wall [16]. Monochorionic pregnancies have a higher incidence of fetal anomalies than dichorionic pregnancies [16].

Following the management of TTTS, an unrelated finding of CPAM added complexity to the pregnancy in our case. CPAM is a rare, benign lung condition with an unknown cause, marked by abnormal lung tissue growth. It occurs in one in 11,000-35,000 births and can be unilateral or bilateral [17]. CPAM is categorized into five types based on sonographic findings in Table 3 [18]. Monitoring involves tracking cyst growth and measuring the CPAM volume ratio (CVR); a CVR greater than 1.6 predicts hydrops, necessitating treatment [19]. The prognosis for antenatally detected CPAM is favorable, with many cases spontaneously regressing in the third trimester or early postnatal period [17]. Regular postnatal follow-up is essential to monitor the cyst's progression and potential regression [17].

**Table 3:** CPAM categorization based on sonographic findings CPAM: congenital pulmonary airway malformation [18]

Type	Description
Type 0	Extremely rare and lethal, with global developmental arrest.
Type 1	Dominant cysts, with or without smaller surrounding cysts.
Type 2	Cysts less than 2 cm, often associated with other abnormalities.
Type 3	Microcysts smaller than 5 mm, usually affecting an entire lobe.
Type 4	Large cysts greater than 10 cm, affecting a single lobe.

## Conclusions

This case illustrates the complexities of managing MCDA twin pregnancies complicated by TTTS. The presence of an eccentric cord could be one of the possible causes for the development of TTTS and also fetal demise. FLP effectively treated TTTS but resulted in the demise of one twin, highlighting the procedure's risks. Successful management of the surviving twin, including monitoring for CPAM, shows the importance of early diagnosis and continuous surveillance. The case also stresses the need for comprehensive care, including patient emotional support. Given the potential for maternal psychological distress, counseling and mental health support are essential. This case shows timely interventions and a multidisciplinary approach can lead to positive outcomes in high-risk pregnancies, emphasizing the importance of effective treatment and holistic care for mothers and fetuses.
